# Effect of Japanese Herbal Kampo Medicine Goreisan on Reoperation Rates after Burr-Hole Surgery for Chronic Subdural Hematoma: Analysis of a National Inpatient Database

**DOI:** 10.1155/2015/817616

**Published:** 2015-10-01

**Authors:** Hideo Yasunaga

**Affiliations:** Department of Clinical Epidemiology and Health Economics, School of Public Health, The University of Tokyo, Tokyo 113-0033, Japan

## Abstract

Goreisan is a herbal Kampo medicine used for treating chronic subdural hematoma (CSDH) in Japan. Experimental studies have suggested that Goreisan exerts a hydrogogue effect, but clinical evidence for the effectiveness of Goreisan in CSDH is currently lacking. Using a national Japanese inpatient database, we examined the association between Goreisan use and reoperation rates after burr-hole surgery for CSDH. We identified 36,020 patients, including 3,889 Goreisan users and 32,131 nonusers. Propensity scores of receiving Goreisan were calculated based on hospital characteristics and patient backgrounds (age, sex, body mass index, activities of daily living, consciousness level, comorbidities, antithrombotic agent use, mannitol infusion, and corticosteroid infusion). One-to-one propensity-score matching created 3,879 pairs of Goreisan users and nonusers. Propensity-matched analysis revealed that Goreisan use was significantly associated with a lower reoperation rate (4.8%) compared with nonuse (6.2%) (risk difference, −1.4%; 95% confidence interval (CI), −2.4% to −0.38%). The number needed to prevent one reoperation was 72 (95% CI, 41–265). Instrumental-variable analysis showed similar results to the propensity-matched analysis. These results suggest that Goreisan use reduced the need for reoperation after burr-hole surgery for CSDH.

## 1. Introduction 

Chronic subdural hematoma (CSDH) is a common type of intracranial hemorrhage, particularly in the elderly. CSDHs are well-delineated collections of blood between the dura matter and arachnoid space. Burr-hole craniotomy is a well-established, first-choice treatment for CSDH; however, studies have shown high recurrence rates of 4%–30% after burr-hole surgery [1–3].

Several treatments have been shown to be useful for treating CSDH. Mannitol or glycerol may be infused as an adjunctive therapy to surgery or as conservative therapy in inoperable patients. Corticosteroid infusion represents another feasible option for the postoperative treatment of CSDH [4, 5].

Goreisan is a Japanese herbal Kampo medicine that has been used to treat asymptomatic CSDH and prevent postoperative recurrence of CSDH in Japan. Experimental studies have suggested that Goreisan exerts a hydrogogue effect [6, 7]. However, previous clinical studies on the use of Goreisan to treat CSDH were small case-series studies with no control group, most of which were published in Japanese journals [8–11]. The clinical effectiveness of Goreisan for the treatment of CSDH thus remains unclear.

In the present study, we examined the association between the use of Goreisan and the need for reoperation after burr-hole surgery for CSDH, using information from a national Japanese inpatient database.

## 2. Methods

### 2.1. Data Source

The present study utilized the Japanese Diagnosis Procedure Combination (DPC) inpatient database. The details of the database have been described elsewhere [12]. In brief, the database includes discharge abstracts and administrative claims data for approximately 7 million inpatients per year, collected from more than 1,000 hospitals, representing approximately 50% of all inpatient admissions to acute-care hospitals in Japan. We obtained inpatient data between July 1, 2010, and March 31, 2013. The database contains the following information: type of hospital (academic or nonacademic); unique identifiers of hospitals; patient age and sex; primary diagnoses, comorbidities at admission, and complications after admission coded according to the International Classification of Diseases, Tenth Revision (ICD-10) codes; drugs and devices used; surgical and nonsurgical procedures; Japan Coma Scale at admission; activity of daily living scores for self-care and mobility (which can be converted to Barthel Index); body weight and height; length of stay; and discharge status. The database also includes information on dates of admission, discharge, surgery, and drug prescribing.

A Japan Coma Scale of 0 indicates alert consciousness; single-digit scores (1, 2, 3) indicate being drowsy but awake without any stimuli; two-digit scores (10, 20, 30) indicate somnolence but being aroused by some stimuli; and three-digit scores (100, 200, 300) indicate coma. The Japan Coma Scale and Glasgow Coma Scale are well correlated [13, 14].

The Barthel Index is a reliable disability scale for stroke patients, which measures the patient's performance in activities of daily life related to self-care (feeding, grooming, bathing, dressing, bowel and bladder care, and toilet use) and mobility (ambulation, transfers, and stair climbing). The maximal score is 100, indicating that the patient is fully independent in terms of physical functioning, while the lowest score is 0, representing a totally dependent, bedridden state [15].

The database also includes estimated total hospitalization costs based on reference prices in the Japanese national fee schedule that determines item-by-item prices for surgical, pharmaceutical, laboratory, and other inpatient services.

The requirement for informed consent was waived because of the anonymous nature of the data. Study approval was obtained from the Institutional Review Board at The University of Tokyo.

### 2.2. Patient Selection

We included patients aged ≥40 years who were hospitalized with CSDH (ICD-10 code, I62.0) as a main diagnosis at admission and who underwent burr-hole surgery within 2 days after admission (i.e., on the day of admission or the following day). We excluded patients younger than 40 years old; patients diagnosed with cerebral infarction, cerebral hemorrhage, or subarachnoid hemorrhage at admission; and patients who used Kampo medicines other than Goreisan. We divided the eligible patients into the following groups: (i) those who started Goreisan within 2 days after surgery (Goreisan users) and (ii) those who did not receive Goreisan (Goreisan nonusers).

### 2.3. Baseline Characteristics

We examined the following patient characteristics: age, sex, Japan Coma Scale at admission, Barthel Index at admission, body mass index (kg/m^2^), comorbidities present at admission, receipt of antithrombotic therapy (including anticoagulants and antiplatelet drugs), mannitol or glycerol infusion started within 2 days after surgery, and corticosteroid infusion within 2 days after surgery.

Body mass index was classified into the following four categories according to the World Health Organization criteria: <18.5 kg/m^2^ (underweight), 18.5–24.9 kg/m^2^ (normal weight), 25.0–29.9 kg/m^2^ (overweight), and ≥30 kg/m^2^ (obesity) [16]. We assessed comorbidities present at admission including malignancy, ischemic heart disease, chronic heart failure, chronic renal failure, liver cirrhosis, pneumonia, urinary tract infection, and sepsis. Hospital volume was defined as the average annual number of patients with CSDH treated at each hospital and was categorized into tertiles (low-, medium-, and high-volume hospitals).

### 2.4. Outcome

The primary outcome was reoperation after burr-hole surgery performed during the same hospitalization or at readmission during the study period. The secondary outcome was total hospitalization costs for both the initial admission and readmissions.

### 2.5. Statistical Analyses

We compared the baseline characteristics between Goreisan users and nonusers using the standardized difference. An absolute standardized difference >10 indicated a significant imbalance in a covariate [17].

One-to-one propensity-score matching was performed between Goreisan users and nonusers. Propensity-score matching addresses the confounding bias inherent in retrospective observational studies, where outcomes may reflect a lack of comparability between treatment groups rather than the effects of the study treatment [18–20]. This approach tries to construct a randomized experiment-like situation where the treatment groups being contrasted are comparable for the observed prognostic factors. Propensity-score matching involves (i) estimation of the propensity score, (ii) matching of patients according to their estimated propensity score, and (iii) comparison of outcomes in matched patients. We estimated the propensity score using a logistic regression model with Goreisan use as the dependent variable and all the baseline characteristics as dependent variables. We calculated the* C*-statistic for evaluating the goodness-of-fit. We then performed one-to-one matching of patients between Goreisan users and nonusers with the closest propensity score within a caliper (≤0.2 of the pooled standard deviation) using the nearest neighbor method. Reoperation rates were compared using Fisher's exact tests and total hospitalization costs were compared using* t*-tests between the groups. We also estimated relative risk, risk difference, and the 95% confidence intervals (CI) for reoperation. The number needed to treat (NNT) was calculated as the reciprocal of the risk difference.

The above results were confirmed using instrumental-variable analysis. Propensity-score analysis and conventional multivariate regression analysis are unable to remove hidden biases caused by unmeasured confounders. Instrumental-variable analysis is another pseudorandomization process that can technically address unmeasured confounders. The key assumptions of the instrumental variable are that (i) it is highly correlated to the treatment assignment, (ii) it is not correlated to any measured or unmeasured patient backgrounds, and (iii) it does not affect patient outcomes except through treatment [21, 22]. In the present study, we defined the percentage of Goreisan use in each hospital as the instrumental variable, calculated by dividing the total number of patients receiving Goreisan by the total number of patients undergoing burr-hole surgery at each hospital. When hospitals showed a strong consistency in terms of Goreisan use, the decision to prescribe it or not was assumed to be made largely independently of individual patients' characteristics. In this scenario, individual Goreisan use depended more on the hospital at which they are treated than on their specific risk factors. Under these conditions, the hospital's preference for Goreisan may act as an instrumental variable, allowing a “natural experiment” for an unbiased estimate of the risk of reoperation, even in the presence of unmeasured confounders. In our instrumental analysis, we only included patients who were admitted to hospitals with at least 10 patients during the study period. We calculated the 90th percentile value for the percentage of Goreisan use, and the instrumental variable was then dichotomized according to the percentage of Goreisan use ≥90th percentile value (Goreisan preference group) or <90th percentile (Goreisan nonpreference group). We used the* F*-test as a weak identification test. An* F*-statistic >20 indicated a strong instrumental variable. We used this instrumental variable to compute the risk difference and its 95% CI for reoperation between Goreisan users and nonusers using a two-stage, least square method that also adjusted for the measured baseline characteristics, for robustness. NNT was calculated as the reciprocal of the risk difference in the instrumental variable.

All statistical analyses were performed using Stata version 13 (StataCorp, College Station, TX), including the “teffects psmatch” procedure for propensity-score matching and the “ivreg2” procedure for instrumental-variable analysis. A two-tailed significance level of 0.05 was used in all statistical analyses.

## 3. Results

We identified 36,020 eligible patients from 797 hospitals, including 3,889 (10.8%) Goreisan users and 32,131 (89.2%) nonusers. One-to-one propensity-score matching created 3,879 pairs (*n* = 7,758). The* C*-statistic for the goodness-of-fit was 0.634. For instrumental-variable analysis, we selected 33,836 patients in 594 hospitals with an average hospital volume of at least 10 patients per year. The* F*-statistic was 27.5, indicating that the percentage of Goreisan use at each hospital was a strong instrumental variable.

Table 1 shows the baseline characteristics of patients with and without Goreisan use in the unmatched and propensity-matched groups. In the unmatched groups, Goreisan was more likely to be used at academic hospitals and in the fiscal year 2012. Goreisan users were less likely to have coma or comorbidities. The baseline characteristics were closely balanced between the two groups after propensity-score matching.

Table 2 shows the reoperation rates and total hospitalization costs in the propensity-matched group. The reoperation rate was significantly lower in Goreisan users than in nonusers (4.8% versus 6.2%; *p* = 0.001). Total hospitalization costs were also significantly lower in Goreisan users than in nonusers (6,428 versus 6,707 US dollars; *p* = 0.030).

Table 3 shows the relative risk, risk differences, and NNT for reoperation rates in Goreisan users with reference to nonusers. In propensity-matched analysis, Goreisan use was significantly associated with a decreased risk difference for reoperation (−1.4%; 95% CI, −2.4% to −0.38%). The estimated NNT was 72. In instrumental-variable analysis, the estimated risk difference was −2.2% (−3.8% to −0.67%) and the NNT was 45.

## 4. Discussion

The present retrospective observational study using a national inpatient database showed a significant reduction in the need for reoperation after burr-hole surgery for CSDH in patients who used Goreisan, compared with those who did not. Total hospitalization costs were also significantly lower in Goreisan users.

Burr-hole surgery is a well-established treatment for CSDH, but blood collection sometimes reoccurs, requiring reoperation [1–3]. Hyperosmolar therapy using mannitol or glycerol is a feasible option for treating brain edema. Several studies have also suggested that corticosteroids may be effective for treating postoperative brain edema [4, 5].

Goreisan, a Japanese herbal Kampo medicine, is a mixture of five herbs including Alismatis Rhizoma, Poria, Polyplus, Atractylodis Lanceae Rhizoma, and Cinnamomi Cortex. Experimental studies have suggested a potential mechanism of hydrostatic modulation caused by Goreisan [6, 7]. Aquaporin-4 is the major water channel for fluid transport in the central nervous system and increases the water permeability of cell membranes. Aquaporin-4 plays an integral role in pathogenesis of brain edema [23]. A previous study showed aquaporin-4 was expressed on CSDH outer membrane [7]. Aquaporin-4 on the CSDH outer membrane may cause excessive fluids into subdural space and contribute to hematoma development. Studies have suggested an inhibitory effect of several herbs on aquaporin-4, including Poria, Polyplus, Atractylodis, and Lanceae Rhizoma, which are ingredients of Goreisan [7, 24]. Goreisan's inhibition of aquaporin-4 on CSDH membrane possibly contributes to reducing volume of CSDH.

Japanese clinicians have reported case series of Goreisan use for CSDH, mostly in Japanese journals [8–11]. However, these studies were limited to small numbers of patients, with no controls or adjustment for patient backgrounds. The present study was therefore the first to demonstrate the effectiveness of Goreisan for reducing the need for reoperation after CSDH surgery in a nationwide clinical setting. This retrospective cohort study used information from a national inpatient database in Japan to examine the impact of Goreisan use on reoperation rates after burr-hole surgery for CSDH, based on a large study population and “pseudorandomized” statistical methods, including propensity-score matching and instrumental-variable analysis. The large dataset enabled us to perform a robust statistical estimation of the effectiveness of Goreisan, with adjustments for measured and unmeasured confounders. Propensity-score matched analysis showed that Goreisan use was associated with a reduction in reoperation rates from 6.2% to 4.8%. Instrumental-variable analysis was performed to confirm these results and showed a similar trend, suggesting that the results were robust.

Several limitations should be acknowledged. First, the database did not include detailed clinical information on laboratory data or computed tomography findings (e.g., size of hematoma). Second, this study was not based on a randomized controlled trial, and its retrospective nature might have been associated with residual confounders, despite the use of robust statistical methods.

## 5. Conclusion

Goreisan use effectively reduced reoperation rates after burr-hole surgery for CSDH, based on a large national database.

## Figures and Tables

**Table 1:** Baseline characteristic of patients with and without Goreisan in unmatched and propensity-matched groups.

	Unmatched groups (*n* = 36,020)			Propensity-matched groups (*n* = 7,758)		
	Goreisan users (*n* = 3,889)	Goreisan nonusers (*n* = 32,131)	Standardized difference	Goreisan users (*n* = 3,879)	Goreisan nonusers (*n* = 3,879)	Standardized difference
Type of hospital, *n* (%)						
Nonacademic	2,997 (77.1)	27,644 (86.0)	19.6	2,997 (77.3)	3,016 (77.8)	1.0
Academic	892 (22.9)	4,487 (14.0)	−19.6	882 (22.7)	863 (22.2)	−1.0
Hospital volume (per year), *n* (%)						
≤22	1,133 (29.1)	9,836 (30.6)	2.6	1,131 (29.2)	1,098 (28.3)	−1.5
23–36	1,283 (33.0)	10,848 (33.8)	1.3	1,275 (32.9)	1,340 (34.5)	2.9
≥37	1,473 (37.9)	11,447 (35.6)	−3.8	1,473 (38.0)	1,441 (37.1)	−1.4
Fiscal year, *n* (%)						
2010	568 (14.6)	7,748 (24.1)	19.2	568 (14.6)	564 (14.5)	−0.2
2011	1,278 (32.9)	12,066 (37.6)	8.0	1,278 (32.9)	1,305 (33.6)	1.2
2012	2,043 (52.5)	12,317 (38.3)	−23.6	2,033 (52.4)	2,010 (51.8)	−1.0
Age (years), mean (SD)	76.5 (10.7)	76.4 (11.1)	−1.4	76.2 (10.7)	76.2 (10.7)	0.0
Sex, *n* (%)						
Male	2,644 (68.0)	21,577 (67.2)	−1.5	2,636 (68.0)	2,618 (67.5)	−0.8
Female	1,245 (32.0)	10,554 (32.8)	1.5	1,243 (32.0)	1,261 (32.5)	0.8
Body mass index (kg/m^2^), *n* (%)						
<18.5	487 (12.5)	4,164 (13.0)	1.1	486 (12.5)	452 (11.7)	−2.2
18.5–22.9	1,712 (44.0)	14,065 (43.8)	−0.4	1,709 (44.1)	1,746 (45.0)	1.6
23.0–24.9	681 (17.5)	5,356 (16.7)	−1.8	681 (17.6)	723 (18.6)	2.3
25.0–29.9	556 (14.3)	4,079 (12.7)	−3.9	550 (14.2)	536 (13.8)	−0.9
≥30.0	57 (1.5)	459 (1.4)	−0.3	57 (1.5)	46 (1.2)	−2.1
Missing	396 (10.2)	4,090 (12.7)	6.4	396 (10.2)	376 (9.7)	−1.4
Barthel Index at admission, *n* (%)						
0	794 (20.4)	7,676 (23.9)	6.8	794 (20.5)	809 (20.9)	0.8
5–45	671 (17.3)	5,679 (17.7)	0.9	670 (17.3)	609 (15.7)	−3.5
50–95	808 (20.8)	6,016 (18.7)	−4.2	804 (20.7)	746 (19.2)	−3.1
100	894 (23.0)	6,740 (21.0)	−4.0	889 (22.9)	991 (25.5)	5.0
Missing	722 (18.6)	6,020 (18.7)	0.4	722 (18.6)	724 (18.7)	0.1
Japan Coma Scale at admission, *n* (%)						
0 (alert)	1,809 (46.5)	14,112 (43.9)	−4.3	1,799 (46.4)	1,898 (48.9)	4.2
1 digit (drowsy)	1,844 (47.4)	15,124 (47.1)	−0.6	1,844 (47.5)	1,747 (45.0)	−4.1
2 digits (somnolence)	190 (4.9)	1,965 (6.1)	4.3	190 (4.9)	185 (4.8)	−0.5
3 digits (coma)	46 (1.2)	930 (2.9)	9.3	46 (1.2)	49 (1.3)	0.6
Comorbidities, *n* (%)						
Malignancy	137 (3.5)	1,359 (4.2)	2.9	137 (3.5)	125 (3.2)	−1.4
Ischemic heart disease	184 (4.7)	1,562 (4.9)	0.5	184 (4.7)	163 (4.2)	−2.2
Chronic heart failure	113 (2.9)	1,057 (3.3)	1.8	113 (2.9)	96 (2.5)	−2.2
Chronic renal failure	53 (1.4)	836 (2.6)	6.9	53 (1.4)	47 (1.2)	−1.1
Liver cirrhosis	22 (0.6)	163 (0.5)	−0.7	22 (0.6)	24 (0.6)	0.5
Pneumonia	27 (0.7)	289 (0.9)	1.8	27 (0.7)	20 (0.5)	−1.9
Urinary tract infection	9 (0.2)	104 (0.3)	1.4	9 (0.2)	12 (0.3)	1.2
Sepsis	2 (0.1)	35 (0.1)	1.6	2 (0.1)	3 (0.1)	0.8
Antithrombotic agent use, *n* (%)	317 (8.2)	2,639 (8.2)	0.2	314 (8.1)	278 (7.2)	−2.9
Mannitol infusion, *n* (%)	104 (2.7)	1,033 (3.2)	2.6	104 (2.7)	84 (2.2)	−2.8
Steroid infusion, *n* (%)	15 (0.4)	243 (0.8)	3.8	15 (0.4)	15 (0.4)	0.0

**Table 2:** Reoperation rates and total hospitalization costs in Goreisan users versus nonusers in propensity-matched groups.

	Goreisan users	Goreisan nonusers	*p*
	(*n* = 3,879)	(*n* = 3,879)	
Reoperation, *n* (%)	187 (4.8)	241 (6.2)	0.001
Total hospitalization costs ($), mean (SD)	6,428 (4,646)	6,707 (6,567)	0.030

SD, standard deviation.

**Table 3:** Relative risks, risk differences, and number needed to treat in reoperation rates comparing Goreisan users with nonusers.

	Relative risk	Risk difference	Number needed to treat
	(95% CI)	(95% CI)	(95% CI)
Propensity-score matching (*n* = 7,758)	0.78 (0.64; 0.93)	−1.4% (−2.4%; −0.38%)	72 (41; 265)
Instrumental-variable analysis (*n* = 33,836)	—	−2.2% (−3.8%; −0.67%)	45 (26; 148)

CI, confidence interval.
